# The Influence of Carbohydrates on the Virulence Potential of *Klebsiella variicola* Isolates

**DOI:** 10.1155/ijm/5403408

**Published:** 2025-09-24

**Authors:** Ana Paula Cardoso Almeida, Laura Fernandes Gonçalves, Rafaella Christina Rocha Moreira da Silva, Ana Flávia Alves Parente, Tatiana Amabile de Campos

**Affiliations:** ^1^Programa de Pós-graduação em Biologia Microbiana, Instituto de Ciências Biológicas, Universidade de Brasília (University of Brasília, UnB), Brasília, Distrito Federal, Brazil; ^2^Departamento de Biologia Celular, Instituto de Ciências Biológicas, Universidade de Brasília, (University of Brasília, UnB), Brasília, Distrito Federal, Brazil

**Keywords:** carbohydrates, galactose, hypermucoviscosity, *Klebsiella variicola*, maltose, sorbitol, virulence

## Abstract

*Klebsiella variicola* is a Gram-negative bacillus belonging to Enterobacteriaceae recognized as an emerging human pathogen in the last years. The species is frequently misidentified as *Klebsiella pneumoniae* by conventional microbiological tests, raising questions about its real prevalence and clinical impact. *K. variicola* virulence is mediated by traits that are expressed in human body niches containing different nutrients, including carbohydrates, that influence their expression. In this way, we analyzed the effect of different carbohydrates on the expression of virulence traits by *K. variicola* isolates. For this approach, three classical strains (cKv15, cKv35, and cKv57) and one hypermucoviscous (HMV) were submitted to growth curve characterization, biofilm production and serum survival assays, siderophores, and *mrk*A (encoding MRK adhesin) RNA quantification. The strains were cultivated in broth containing specific carbohydrates as the sole carbon source to perform the assays. Among all carbohydrates tested, sorbitol, galactose, and maltose were the most effective in promoting biomass production in biofilm for the *K. variicola* classical strains (cKv). Additionally, bacterial incubation in these carbohydrates resulted in the production of siderophores by all strains (cKv and HMV). Notably, cKvs cultivated in all carbohydrates tested survived and proliferated in human serum, while also producing high concentrations of siderophores and biofilm biomass. Except for siderophore production, HMV did not present any virulence trait tested (biofilm production, serum survival, and *mrk*A expression). However, its growth in media supplemented with galactose promoted serum survival. These observations indicate cKv isolates were able to use sorbitol, galactose, and maltose for rapid proliferation and to express determinants associated with bacterial colonization (as biofilm production, siderophores, and serum survival). The hypermucoviscosity of HMV did not promote biofilm and siderophore production. For this strain, galactose promoted survival in human serum. Altogether, the results highlight the role of galactose in promoting virulence in *K. variicola.*

## 1. Introduction

*Klebsiella variicola* is an enterobacterium that has gained attention in recent years as an emerging human pathogen, associated with severe infections and multiple resistance to antibiotics [1–3]. Although it is commonly identified as *Klebsiella pneumoniae* in conventional microbiological tests, recent studies reveal that *K. variicola* exhibits distinct phenotypic and genotypic characteristics, raising questions about its actual prevalence and clinical impact. It is a ubiquitous species, being isolated in a wide range of environments, from plants to humans and animals [4]. In human infections, *K. variicola* is prevalent in nosocomial infections, mainly among immunocompromised or trauma patients, which reinforces the need for improved diagnostic methods to differentiate it from other *Klebsiella* species [4].

The pathogenicity of *K. variicola* is determined by various virulence factors that help it to evade the host's innate immunity. Among these factors, lipopolysaccharides, capsular polysaccharides, siderophores, and fimbriae stand out, playing crucial roles in the bacterium's ability to colonize and invade host tissues [5]. These components are common among *Klebsiella* species, but their contribution to *K. variicola* pathogenicity in severe infections is not precisely described. In parallel, *K. variicola* antimicrobial resistance harboring strains increase the risk of mortality due to infection treatment difficulties.

The metabolism of different carbon sources is a key factor to the pathogenicity and virulence of enterobacteria. These bacteria's ability to adjust gene expression in response to available nutrients in the environment is directly associated with their pathogenic behavior [6, 7]. Studies indicate that the type of carbon source available can modulate the expression of virulence-related genes in several bacterial species. For example, the presence of glucose has been associated with increased antimicrobial resistance and the formation of protective structures, facilitating defense against the host's immune system and colonization of surfaces in hospital environments [6, 8].

Carbohydrates as carbon sources have demonstrated specific impacts on the regulation of virulence factors, influencing the expression of components crucial for adhesion and tissue invasion [7]. Understanding how these carbon sources affect the pathogenic behavior of enterobacteria can be useful for developing strategies to control and prevent infections [6–8]. In this study, we analyzed the effect of different carbohydrates available in the gastrointestinal tract, where *Klebsiella* commonly colonizes, on the expression of virulence traits by *K. variicola* isolates (classical and hypermucoviscous [HMV]) to explore their roles in bacterial pathogenicity.

## 2. Material and Methods

### 2.1. Bacterial Isolates and Growth Conditions

*K. variicola* isolates were obtained from different bacterial cultures: urine (named cKv15), blood (cKv35), and catheter (cKv57). All strains were isolated at a tertiary scholar hospital (Hospital Universitário de Brasília, HUB/UnB) and were previously characterized as multidrug resistant [1]. *K. variicola* HMV was used as control by presenting hypermucoviscosity phenotype [7]. To perform the assays, the strains were cultured in LB (Kasvi), M9 minimal medium (Sigma-Aldrich), and M9 add 2% of D-glucose, D(-)fructose, lactose, D(-)sorbitol, D(+)maltose, D(+)galactose, and mannose (Sigma-Aldrich) when convenient. All strains were maintained at −80°C in LB with 15% glycerol. The incubation temperature used is described below. The strains cKv15, cKv35, and cKv57 were also referred to as classical (cKv) as they do not present hypermucoviscosity.

### 2.2. Growth Curve

The growth curve was carried out to determine the multiplication capacity of each strain using different carbon sources. For this purpose, bacterial strains were cultured in LB and in M9 medium supplemented with 2% of each carbon source tested (D-glucose, D(-)fructose, lactose, D(-)sorbitol, D(+)maltose, D(+)galactose, and mannose) at 37°C, until their OD_600_ value reached 0.1 [9]. OD_600_ values were measured on the Pharmacia LKB Ultrospec III Spectrophotometer (Pharmacia, LKB, DG Apeldoorn, Netherlands). Then, 50 *μ*L of the bacterial suspension was added to 150 *μ*L of its respective culture medium in a 96-well plate. The plate was then incubated at 37°C for 24 h in aerobic conditions, with OD_600_ values measured every 30 min on the Spectra Max M3 (Molecular Devices, LLC, San Jose, California, United States). All growth curves were performed individually in each carbon source tested in triplicate. Parameters such as growth rate (*μ*) and generation time (*g*) were determined as follows: *μ* = ln(OD_2_) − ln(OD_1_)/*t*_2_ − *t*_1_ where OD_2_ is the OD_600nm_ value at 8 h determined as the mid log phase time and OD_1_ is the OD_600nm_ value at 0 h, *t*_2_ is the time of 8 h and *t*_1_ is 0 h, and generation time *g* = ln(2)/*μ*.

### 2.3. Biofilm Production

The quantification of biofilm production was done by optical density as described by [10]. The assay was performed in triplicate and in three biological replicas on polystyrene surface plates incubated in 37°C for 24 h and for 5 days. The biofilm production was determined in LB growth, M9, and M9 containing each carbon source tested (as described before).

### 2.4. Siderophore Production

Quantification of siderophore production by isolates was carried out according to [11] using the Chrome Azurol S (CAS) technique. This method uses an iron dye complex that changes color when the iron is removed. Siderophores have a greater affinity for iron, so when the siderophore sequesters iron, the color suspension changes from blue to yellow.

The CAS assay solution was prepared by mixing 1.5 mL of 2 mM CAS solution with 1.5 mL of 1 mM Fe^3+^ solution. To this mixture, 50 mL of 1.2 mM hexadecyltrimethylammonium bromide (HDTMA) was added, followed by 30 mL of 0.5 M piperazine buffer (pH 5.6). The final solution retained a deep blue color, characteristic of the CAS-Fe^3+^ complex.

For the siderophore detection assay, 100 *μ*L of 10× concentrated microbial culture supernatant was mixed with 100 *μ*L of CAS assay solution. If siderophores were present, they chelated Fe^3+^, disrupting the CAS-Fe^3+^ complex and resulting in a color change. The degree of iron removal was quantified spectrophotometrically at 630 nm.

The concentrated supernatant was obtained by cultivating the bacterial isolates in M9 minimal medium that had been iron-depleted using 1% (*w*/*v*) Chelex 100 resin. After 1 h of agitation, the Chelex beads were removed by filtration. *K. variicola* strains were cultivated in 10 mL of iron-chelated M9 minimal medium for 24 h. The bacterial cells were then removed by centrifugation at 12,000 rpm for 10 min at 4°C, followed by filtration through a 0.22-*μ*m membrane filter. The resulting supernatant was freeze-dried and resuspended in 1 mL of ultrapure water prior to use in the assay.

### 2.5. Human Serum Bacterial Survival

The survival rate of the bacterial strains survival in human serum was as determined by [12]. Bacterial suspensions previously cultured in each condition tested were diluted to OD_600nm_ 0.3 (approximately 10^7^ CFU/mL), pelleted (4 min—12,000*g*), resuspended in 550 *μ*L of human serum, and incubated at 37°C under 60 rpm. At determined time points (0, 30, 60, and 120 min), 10 *μ*L of each suspension was collected, diluted to 10^−4^ and plated on MacConkey agar (Kasvi). After 18 h of incubation at 37°C, the CFUs observed were counted.

### 2.6. Qt-RT PCR

The Qt-RT PCR was done to determine the expression of *mrk*A. For this approach, the strains were cultivated in each condition tested until OD_600nm_ 0.3. Total RNA was extracted from each bacterial suspension, preserved in phenol at −80°C, and purified using the ReliaPrep miRNA Cell and Tissue Miniprep System (Promega). RNA quality was assessed by electrophoresis in 1% agarose gels, and the RNA concentration was determined by NanoDrop One spectrophotometer (Thermo Fisher Scientific, Waltham, Massachusetts, United States). One microgram of RNA was used for cDNA synthesis, using the SuperScript IV Reverse Transcriptase kit (Invitrogen, Thermo Fisher Scientific, Waltham, Massachusetts, United States), according to the manufacturer's instructions.

The analysis of gene expression was conducted using the iTaq Universal SYBR Green Kit (Bio-Rad, Hercules, California, United States). Each qPCR reaction was conducted in a final volume of 10 *μ*L, containing 2 *μ*L of a 1:20 dilution of each cDNA stock, 0.2 *μ*M of each primer, and 5 *μ*L of the iTaq Universal SYBR Green Kit. PCR amplifications were carried out on an ABI StepOne Real-Time PCR thermocycler (Applied Biosystems, Thermo Fisher Scientific, Waltham, Massachusetts, United States) according to the following parameters: an initial two-step phase of 50°C for 2 min and 95°C for 10 min, 40 cycles of denaturation at 95°C for 15 s, and primer annealing and extension at 60°C for 60 s. Three biological and three technical replicates were included for each gene. Melting temperatures (Tm) were determined using the StepOne Software v2.3 (Applied Biosystems, Thermo Fisher Scientific, Waltham, Massachusetts, United States). The *mrk*A expression was determined by comparison of *rrsH* gene amplification. The primers used for amplification are listed as follows: *rrs*H-fw (5⁣′-3⁣′) GACGATCCCTAGCTGGTCTG; *rrs*H-rv (5⁣′-3⁣′) GTGCAATATTCCCCACTGCCT; *mrk*A-fw (5⁣′-3⁣′) GCGGATACTTACCTGAAACC; *mrkA-*rv (5⁣′-3⁣′) TGCTTACGTCATCCTGTTTAG.

### 2.7. Statistical Analysis

One-way ANOVA and Kruskal–Wallis test were conducted to determine the significance of groups analyzed by using GraphPad Prism v. 10.0 (GraphPad Software 2023).

## 3. Results

### 3.1. Growth Dynamics of *K. variicola* in Single-Carbon-Source Media

The growth rate (*μ*) and generation time (*g*) of *K. variicola* strains were evaluated after incubation in different broths to assess the impact of specific carbohydrates on bacterial fitness. Strains were cultivated at 37°C in LB, M9 salts, or M9 supplemented with a single carbon source: glucose, fructose, galactose, lactose, maltose, mannose, or sorbitol. Since glucose is considered the preferred carbon source in many model species, it was employed as a comparative control.

**Figure 1:**
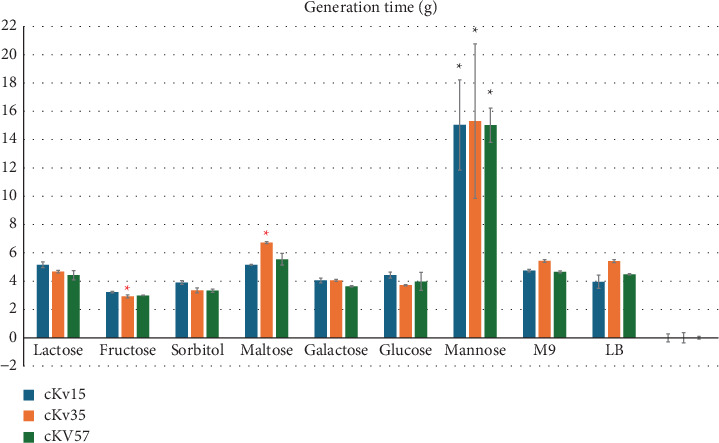
Generation time in hours (*g*) presented by *Klebsiella variicola* strains under different culture conditions. The strains were statically cultivated at 37°C in medium containing different carbohydrates as the unique carbon source (M9 + glucose, M9 + fructose, M9 + galactose, M9 + lactose, M9 + maltose, M9 + mannose, and M9 + sorbitol). The strains were also cultivated in LB and M9 salts. Statistical significance (*p* < 0.05) of values compared to bacterial growth in mannose was indicated by ∗. Red asterisks (∗) show the statistical significance (*p* < 0.05) of values of fructose growth compared to maltose.

Among the tested carbohydrates, mannose led to the longest generation times in all strains, indicating reduced bacterial fitness (Table S1). Otherwise, glucose, sorbitol, fructose, galactose, and maltose provided the highest *μ* values for strains (Supporting Information). The shortest generation times were observed with fructose. However, these values were significantly different only in comparison to mannose for all strains and to maltose for cKv35 (Figure 1).

### 3.2. Effect of Individual Carbohydrates on Biofilm Development

The use of those carbohydrates in biofilm production in 24 h was tested (Table 1). Since growth in sorbitol, maltose, and galactose was associated with both short generation time and high biofilm biomass, these carbohydrates were selected to do the subsequent assays (mature biofilm, siderophore production, serum survival, and *mrkA* expression). Glucose was used as a control. Additionally, a HMV strain was incorporated into the subsequent analyses to allow comparison with the classical strains (cKv15, cKv35, and cKv57).

**Figure 2:**
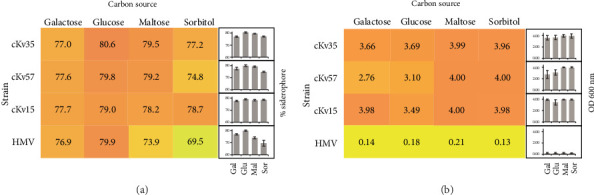
Heatmap showing siderophore and biofilm production by classical (cKv15, cKv35, and cKv57) and HMV *Klebsiella variicola* strains cultured at 37°C in broth containing different carbohydrates (galactose, glucose, maltose, or sorbitol) as the sole carbon source. (a) Siderophore production: the strains were cultured at 37°C in broth containing different carbohydrates as the sole carbon source. (b) Biofilm production determined by bacterial biomass quantified after 120 h (5 days) of the strain's incubation at 37°C in polystyrene plates containing specific carbohydrates as the unique carbon source. OD600nm: optical density. The assays were done in biological and repetition's triplicates. Biofilm production by cKv strains was significantly higher (*p* < 0.05) than those observed by HMV.

To assess the impact of these carbohydrates on the biomass of mature biofilm, surface-associated growth was quantified over a period of 5 days. This experiment revealed that strains grown in glucose, galactose, maltose, and sorbitol exhibited significant biofilm formation (Figure 2b), highlighting the pivotal role of these carbohydrates in promoting biofilm development. Interestingly, the HMV strain produced the lowest biofilm biomass under all tested conditions compared to the classical strains (Figure 2b).

### 3.3. Impact of Individual Carbohydrates on Siderophore Production

Moreover, the production of siderophores, which are critical for iron acquisition and bacterial virulence, was examined following the protocol outlined by Payne [11]. All tested strains exhibited similar siderophore production under every condition tested, indicating that siderophore production is a consistent trait among the *K. variicola* isolates analyzed (Figure 2a). However, it was observed that there was a slight but statistically significant decrease in siderophore production under different carbon source conditions: maltose and sorbitol for HVM, sorbitol and galactose for cKv35 and cKv57. These findings suggest that carbon sources can interfere with *K. variicola* infection dynamics, since iron availability and uptake are key determinants of bacterial adaptation and virulence in *Klebsiella* spp.

### 3.4. Influence of Sole Carbohydrate Sources on the Serum Resistance

**Figure 3:**
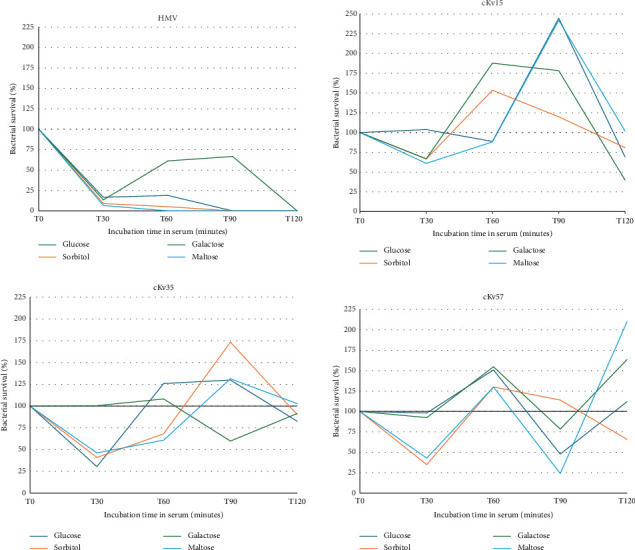
Survival capacity to human serum shown by classical (cKv15, cKv35, and cKv57) and hypermucoviscous (HMV) *Klebsiella variicola* strains. The strains, previously cultivated in broth containing specific carbohydrates (glucose, sorbitol, galactose, or maltose) as a unique carbon source, were incubated in human serum at 37°C. Aliquots from the serum suspension were plated in MacConkey agar after 30, 60, 90, and 120 min. Colony-forming units were counted to determine bacterial survival. The assays were done in biological and repetition triplicates.

Serum survival assay was conducted to assess the resilience of bacterial strains to human serum (Figure 3). All cKvs exhibited survival and proliferation in human serum across all tested carbohydrates (Figure 3). Interestingly, HMV strain showed serum resistance only when incubated in galactose, indicating that the HMV phenotype alone does not confer serum resistance. Furthermore, bacterial growth in galactose enhanced survival against serum-mediated killing in both classical strains and the HMV strain when compared to the other conditions tested (Figure 3).

### 3.5. Influence of Different Carbon Sources on *mrkA* Gene Expression in *K. variicola*

**Figure 4:**
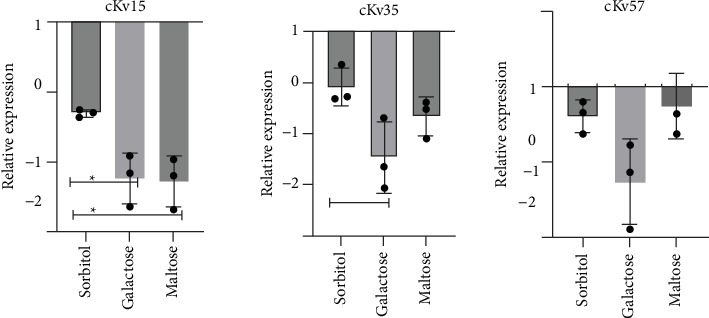
Relative quantification of *mrk*A RNA expression by classical (cKv15, cKv35, and cKv57) *Klebsiella variicola* strains. The strains were previously cultivated in broth containing specific carbohydrates as a unique carbon source for mRNA extraction. The *rr*H housekeeping gene was used to normalize. The assays were done in biological and repetition triplicates. Statistical significance (*p* < 0.05) is indicated by ∗.

The expression of *mrk*A, a gene encoding an adhesin important to initial colonization by bacterial biofilm [13], was investigated through mRNA quantification by qPCR. Transcripts were detected only when the strains were grown in sorbitol, galactose, and maltose (Figure 4). Except for cKv57, the *mrk*A expression was higher in sorbitol than in galactose and maltose. The *mrk*A transcript was not detected in the HMV strain in any condition tested.

## 4. Discussion

Bacterial survival depends on the successful colonization of diverse and dynamic environments, both inside and outside the host, requiring metabolic adaptation to available carbon sources [14]. In this way, we evaluated the effect of different carbohydrates as unique carbon sources on biofilm and siderophore production, survival in human serum, and adhesin expression by one HMV and three classical *K. variicola* strains (cKv15, cKv35, and cKv57).

Our results showed that all strains used in an efficient way glucose, sorbitol, fructose, sucrose, and galactose as carbon sources by providing high growth rates and low generation time, highlighting their metabolic flexibility (Figure 1). In the gastrointestinal tract, where multiple carbon sources are present, this flexibility is crucial, as the ability to cometabolize these substrates can influence the bacterium's virulence and transmission [14, 15].

Predominantly found in human body fluids and tissues and primarily involved in protein glycosylation rather than in carbohydrate metabolism, mannose incubation resulted in the slowest growth rate for all classical strains tested (cKv15, cKv35, and cKv57). These findings indicate reduced efficiency in supporting *K. variicola* replication, suggesting the restricted contribution as an energy source and potential regulatory effects on the gut microbiome [16]. Given that classical *K. variicola* (cKv) is found in human guts, mannose may limit its replication.

Galactose, maltose, and sorbitol yielded the highest biomass in early and mature biofilm by the cKv isolates (Table 1 and Figure 2a), suggesting their role in promoting the increase of the biomass since its early formation. Large bacterial biomass in biofilms increases the infectious potential, as it promotes colonization of surfaces such as catheters, enabling bacterial entry into the body and causing infections like bacteremia [17]. The polysaccharide matrix of biofilm limits the access of host antibodies and antimicrobial peptides, reducing the effectiveness of the complement system and phagocytosis [18].

The HMV strain was isolated from a root canal in the oral cavity of a young woman and represents the first *K. variicola* reported presenting hypermucoid capsule and causing a primary endodontic infection [7]. HMV strains often spread from the infection site to other areas, complicating removal and treatment [19]. However, HMV did not produce strong biofilm under any condition tested (Figure 2a), suggesting that hypermucoviscosity of this strain is unrelated to biofilm formation and may be associated with other biological processes during infection.

Siderophores are crucial for bacterial survival in host fluids. In *Klebsiella* spp., iron uptake is a key determinant of bacterial adaptation and virulence [20]. Thus, it was expected that high levels of siderophores would be observed in all tested conditions (Figure 2b). However, their increased production in specific carbohydrates suggests that certain carbon sources can intensify siderophore synthesis in *K. variicola*, potentially complicating infections since iron is vital for survival, pathogenicity, and central metabolic processes.

Serum survival reflects the virulence potential as the complement system in human serum promotes bacterial clearance, either by increasing the activity of phagocytic cells or by promoting bacterial lysis through the formation of the membrane attack complex [21]. Our results showed that hypermucoviscosity is not associated with bacterial survival to serum killing (Figure 3). HMV survived in serum only when galactose was provided as a carbon source (up to 120 min of incubation) (Figure 3). These findings associated with the serum survival presented by cKv incubated in galactose suggest a role of this carbohydrate in promoting *K. variicola* virulence. Previously reported of galactose in enhancing bacterial virulence was described in pneumococcal infections in mice [22]. cKvs survived in all tested conditions, indicating higher virulence potential than HMV. This finding was surprising, given that the polysaccharide capsule is recognized as a protective factor for *Klebsiella* against complement-mediated killing [21, 23].

Since *mrk*A gene encodes an adhesin crucial for biofilm production in *K. pneumoniae* [13], its expression was evaluated under biofilm-promoting conditions. The absence of *mrkA* transcripts under bacterial incubation in glucose, together with their low expression under incubation in other carbohydrates tested, was maybe explained by regulation mediated by the CRP–cAMP system. In *K. pneumoniae*, this global regulatory network modulates type 3 fimbrial gene expression according to carbon source availability. The phosphotransferase system (PTS) senses preferred carbohydrates such as glucose and inhibits adenylate cyclase activity, thereby lowering intracellular cAMP levels. Reduced cAMP limits CRP activation, decreasing *mrkA* transcription [24].

Our findings also suggest that *mrkA* is not the main colonization factor under the tested conditions, with other adhesins, fimbriae, or factors likely contributing more substantially. Nonetheless, the elevated expression in galactose indicates the impact of this carbohydrate on the virulent potential of the strains, as evidenced by serum survival, biofilm, and siderophore production.

## 5. Conclusion

Among all carbohydrates tested, sorbitol, galactose, and maltose were the most effective at promoting biomass production in biofilm for the *K. variicola* classical strains (cKv). Additionally, bacterial incubation in these carbohydrates resulted in the production of siderophores by all strains (classical and HMV). Notably, all strains incubated in galactose survived and proliferated in human serum, while classical strains also produced high concentrations of siderophores and biofilm biomass. These observations indicate that galactose is a significant carbon source that enhances the expression of virulence traits in the tested cKv isolates.

## Figures and Tables

**Table 1:** Biofilm production by *Klebsiella variicola* strains incubated at 37°C in different culture media for 24 h. The biofilm production is represented by the mean ± standard deviation (SD) value of the bacterial biomass quantified by spectrophotometry in OD_570nm_.

**Medium**	**Strains**		
	**cKv15**	**cKv35**	**cKv57**
M9 + 2%lactose	0.752 ± 0.30	1.147 ± 0.39	1.174 ± 0.27
M9 + 2%fructose	0.944 ± 0.2	1.160 ± 0.45	0.880 ± 0.23
**M**9 + 2%**sorbitol**	1.22 ± 0.49	1.424 ± 0.43	1.586 ± 0.48
**M**9 + 2%**maltose**	1.259 ± 0.47	1.396 ± 0.71	1.371 ± 0.58
**M**9 + 2%**galactose**	1.500 ± 0.55	1.4189 ± 0.68	1.054 ± 0.72
M9 + 2%glucose	0.4137 ± 0.15	0.814 ± 0.17	0.678 ± 0.13
M9 + mannose	0.809 ± 0.08	1.298 ± 0.44	1.191 ± 0.13^b^
Luria Bertani (LB)^a^	0.247 ± 0.13	0.267 ± 0.11	0.199 ± 0.07

*Note:* M9 medium does not contain organic compounds. The bold entries showed the highest biofilm production.

Abbreviation: SD, standard deviation.

^a^Biomass produced was significantly lower than in all other conditions.

^b^cKv57 also presented high biofilm production in mannose growth.

## Data Availability

The data that supports the findings of this study are available in the supporting information of this article.

## Supporting Information

Supporting Information Additional supporting information can be found online in the Supporting Information section. Growth curve and biofilm production by *Klebsiella variicola* isolates. The growth curve was determined by OD_600nm_ quantification during incubation of the isolates at 37°C in broth containing specific carbohydrates as the unique carbon source (glucose, fructose, galactose, lactose, maltose, mannose, or sorbitol) for 24 h. Parameters such as growth rate (*μ*) and generation time (*g*) were determined as follows: *μ* = ln(OD_2_) − ln(OD_1_)/*t*_2_ − *t*_1_ where OD_2_ is the OD_600nm_ value at 8 h determined as the mid log phase time and OD_1_ is the OD_600nm_ value at 0 h, *t*_2_ is the time of 8 h and *t*_1_ is 0 h, and generation time *g* = ln(2)/*μ*. The biofilm production was determined by OD_600nm_ quantified after the incubation of the isolates at 37°C in polystyrene plates containing specific carbohydrates as the unique carbon source (galactose, maltose, glucose, or sorbitol) for 5 days.
